# A generalized deep learning model for heart failure diagnosis using dynamic and static ultrasound

**DOI:** 10.2478/jtim-2023-0088

**Published:** 2023-07-05

**Authors:** Zeye Liu, Yuan Huang, Hang Li, Wenchao Li, Fengwen Zhang, Wenbin Ouyang, Shouzheng Wang, Zhiling Luo, Jinduo Wang, Yan Chen, Ruibing Xia, Yakun Li, Xiangbin Pan

**Affiliations:** Department of Structural Heart Disease, National Center for Cardiovascular Disease, China and Fuwai Hospital, Chinese Academy of Medical Sciences and Peking Union Medical College, Beijing 100037, China; National Health Commission Key Laboratory of Cardiovascular Regeneration Medicine, Beijing 100037, China; Key Laboratory of Innovative Cardiovascular Devices, Chinese Academy of Medical Sciences, Beijing 100037, China; National Clinical Research Center for Cardiovascular Diseases, Fuwai Hospital, Chinese Academy of Medical Sciences, Beijing 100037, China; State Key Laboratory of Cardiovascular Disease, Fuwai Hospital, National Center for Cardiovascular Diseases, Pediatric Cardiac Surgery Center, Fuwai Hospital, Chinese Academy of Medical Sciences and Peking Union Medical College, Beijing 100037, China; Zhengzhou University People’s Hospital, Henan Provincial People’s Hospital, Huazhong Fuwai Hospital, Pediatric Cardiac Surgery, Zhengzhou 450000, Henan Province, China; Department of Echocardiography, Fuwai Yunnan Cardiovascular Hospital, Kunming 650000, Yunnan Province, China; University of Science and Technology of China, School of Cyber Science and Technology, Hefei 230000, Anhui Province, China; Department of Medicine I, University Hospital Munich, Ludwig-Maximilians-University Munich, Munich D-80539, Germany; Laboratory of Experimental Intensive Care and Anesthesiology, Academic Medical Center, Amsterdam 1105 AZ, The Netherlands

**Keywords:** echocardiography, heart failure, deep learning, artificial intelligence, multi-center cross-sectional study

## Abstract

**Objective:**

Echocardiography (ECG) is the most common method used to diagnose heart failure (HF). However, its accuracy relies on the experience of the operator. Additionally, the video format of the data makes it challenging for patients to bring them to referrals and reexaminations. Therefore, this study used a deep learning approach to assist physicians in assessing cardiac function to promote the standardization of echocardiographic findings and compatibility of dynamic and static ultrasound data.

**Methods:**

A deep spatio-temporal convolutional model r2plus1d-Pan (trained on dynamic data and applied to static data) was improved and trained using the idea of “regression training combined with classification application,” which can be generalized to dynamic ECG and static cardiac ultrasound views to identify HF with a reduced ejection fraction (EF < 40%). Additionally, three independent datasets containing 8976 cardiac ultrasound views and 10085 cardiac ultrasound videos were established. Subsequently, a multinational, multi-center dataset of EF was labeled. Furthermore, model training and independent validation were performed. Finally, 15 registered ultrasonographers and cardiologists with different working years in three regional hospitals specialized in cardiovascular disease were recruited to compare the results.

**Results:**

The proposed deep spatio-temporal convolutional model achieved an area under the receiveroperating characteristic curve (AUC) value of 0.95 (95% confidence interval [CI]: 0.947 to 0.953) on the training set of dynamic ultrasound data and an AUC of 1 (95% CI, 1 to 1) on the independent validation set. Subsequently, the model was applied to the static cardiac ultrasound view (validation set) with simultaneous input of 1, 2, 4, and 8 images of the same heart, with classification accuracies of 85%, 81%, 93%, and 92%, respectively. On the static data, the classification accuracy of the artificial intelligence (AI) model was comparable with the best performance of ultrasonographers and cardiologists with more than 3 working years (P = 0.344), but significantly better than the median level (*P* = 0.0000008).

**Conclusion:**

A new deep spatio-temporal convolution model was constructed to identify patients with HF with reduced EF accurately (< 40%) using dynamic and static cardiac ultrasound images. The model outperformed the diagnostic performance of most senior specialists. This may be the first HF-related AI diagnostic model compatible with multi-dimensional cardiac ultrasound data, and may thereby contribute to the improvement of HF diagnosis. Additionally, the model enables patients to carry “on-the-go” static ultrasound reports for referral and reexamination, thus saving healthcare resources.

## Introduction

Heart failure (HF) is a chronic progressive condition that imposes a heavy disease burden and affects nearly 24 million people worldwide, with a 5-year mortality rate of approximately 50%.^[1]^ Therefore, accurate diagnosis, longterm observation, and a “before-and-after” comparison of HF conditions are crucial. One critical indicator is the cardiac ejection fraction (EF, output per beat as a percentage of the end-diastolic volume of the ventricle).^[1]^ Cardiac ultrasonography is the most commonly used clinical tool for the diagnosis of HF. However, it relies on the operator’s experience; hence, the examination results may not truly reflect the patient’s condition.^[2,3]^ Additionally, training specialized ultrasonographers or cardiologists who can competently perform the examination does not adequately meet the growing demand for testing.^[4,5]^ Patients in remote areas often need to travel to cities with concentrated medical resources for medical care because of the uneven distribution of medical resources across regions.^[6,7]^ The difficulty of communicating, carrying, and displaying dynamic ultrasound images across regions as static images could prevent a comprehensive understanding of the patient’s disease progression.

To the best of our knowledge, studies reported to date that use artificial intelligence (AI) methods to assist in the diagnosis of HF have primarily focused on dynamic ultrasound.^[8, 9, 10, 11]^ Studies that classify static ultrasound images or are compatible with dynamic and static ultrasound data are still lacking. Therefore, the aim of this study is to demonstrate the feasibility of this approach to integrate multi-dimensional data and to provide more ideas for subsequent studies. Overall, the findings improve the accuracy of cardiac ultrasound, and help physicians and patients to better manage the progression of HF.

## Materials and Methods

### Data sources

Three datasets included in this study were the EchoNet-Dynamic dataset published by David Ouyang *et al*.^[8]^ in *Nature*, the Cardiac Acquisitions for Multi-structure Ultrasound Segmentation (CAMUS) dataset published by Sarah Leclerc *et al*.^[9]^ in *IEEE Transactions on Medical Imaging*, and a local dataset from the National Cardiovascular Center of China.

The EchoNet-Dynamic dataset consists of 10030 echocardiographic videos labeled with EFs and was used primarily for model training. The data are publicly available and were obtained after signing the “Stanford University School of Medicine EchoNet-Dynamic Dataset Research Use Agreement.” This study was approved by the Stanford University Institutional Review Board and data privacy was reviewed through a standardized workflow by the Center for Artificial Intelligence in Medicine and Imaging, and the University Privacy Office.

In total, 450 ECG videos labeled with EFs were included in the CAMUS dataset, and divided into training and validation sets in the ratio of 8:2. These videos were then randomly segmented into 8976 still images, which were primarily used for model training, validation, and expert comparison. The data are publicly available after registration on the online platform (https://www.creatis.insa-lyon.fr/Challenge/camus/index.html).

The local dataset was derived from intraoperative ultrasound videos of five patients who underwent ultrasound-guided or ultrasound-assisted interventional cardiac procedures (structural heart disease) at Fuwai Hospital between January 2018 and June 2019. The EF values in each video were examined by three registered sonographers with more than 3 years of experience, who confirmed the values only if all three of them agreed. This procedure aimed to validate the model’s ability to classify independent dynamic ultrasound data. The Ethics Committee of Fu Wai Hospital, Chinese Academy of Medical Sciences, approved this study (Approval No. 2022-1672) and waived the requirement for patient consent. Information, such as the time of acquiring the ultrasound image and patient name, was removed from the cardiac ultrasound image data before modeling was performed. The data desensitization process was performed in strict accordance with the requirements of the ethics committee to protect patient privacy.

Additionally, t-SNE visualization of the EchoNet-Dynamic and CAMUS datasets was performed to address the difficulty in classification.^[12]^

### Dynamic recognition capability of the model

**Figure 1:**
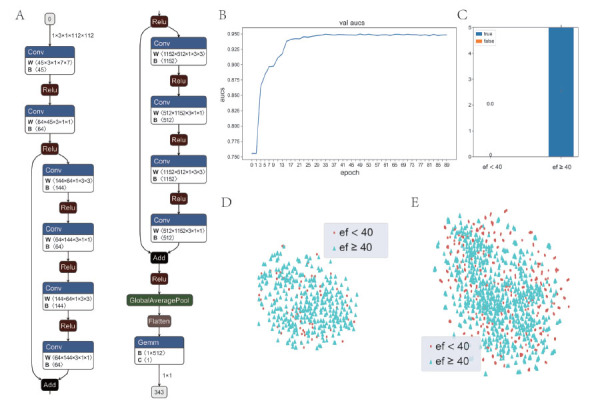
Partial structure of the model, data visualization, and training effect on dynamic data. A. Structure diagram of the input and output of the model. B. Variation of AUC of the model with the number of trainings on the dynamic dataset. C. Classification effect of the model on the local dataset. D. Visualization of the CAMUS dataset. E. Visualization of the EchoNet-Dynamic dataset.

We examined the model’s performance on other independent datasets based on the data and code shared by David Ouyang *et al*.^[8]^ The model could not be applied to static images; therefore, 3D convolution, regression training, and a classification application based on the r2plus1d model were used to enhance the migration application of the model.^[13]^ First, the regression output bias parameter of the model was set to a classification threshold value of 40 (EF) and trained using dynamic data. Subsequently, the category of the obtained EF scores was determined using the classification threshold. Additionally, the published model sampled video sequences of a specific length at successive intervals so that the input dynamic video frames remained in a particular temporal order. However, this approach did not yield good results in migration applications; primarily, static migration applications. Furthermore, a random sampling approach was used for input data loading to eliminate the influence of the before-and-after relationship of video frame images on the results, that is, a specific number of video frame images were randomly selected from the video and combined as dynamic inputs. Only the input and output model structures are shown in Figure 1A because of the large scale of the model. The details of the complete model are provided in the Appendix.

The model is referred to as “r2plus1d-Pan” for ease of designation. The improved model was trained on EchoNet-Dynamic data and validated on local dynamic data.

## Static recognition capability of the model

Data were allocated and models were trained separately using two schemes to explore the ability of the model to be applied in migration applications on static datasets.

r2plus1d-Pan1: The CAMUS dataset was not involved in training and the model was applied directly to perform the classification tasks.

r2plus1d-Pan2: 80% of the CAMUS dataset was involved in training. Additionally, the model was applied directly to 20% of the validation set and compared with human experts.

1, 2, 4, and 8 static images from the same ECG were randomly used as input while the classification task was performed to observe the classification accuracy of the two models.

### Comparison with human experts

In total, 16 ultrasound or cardiology specialists were recruited from the National Cardiovascular Center Beijing headquarters, and Zhengzhou and Kunming branches, and divided into three groups: less than 1 year of relevant experience, 1 to 3 years of relevant experience, and more than 3 years of relevant experience. 3, 3 and 3 ultrasonographers and 3, 3 and 1 cardiologist from each group, respectively, participated in the man-machine comparison study. Because the structure of the model does not allow for the plotting of ROC curves, the interpretation results of the AI model versus human experts for inputs 1, 2, 4, and 8 images were presented as scatter plots. To place human experts and AI models on an equal footing for comparison, we provided the training dataset from the CAMUS dataset to human experts to learn from them.

### Statistics

Deep spatio-temporal convolutional models were built using Python software (3.9). Consecutive values were compared using the Student’s *t*-test or Mann–Whitney U test. All comparisons were two-sided, and a *P* value < 0.05 was considered significant. Random sampling was implemented using the NumPy package random function.

## Results

### Data distribution and dynamic recognition capability of the model

The CAMUS dataset (Figure 1D) and EchoNet-Dynamic dataset (Figure 1E) were visualized separately. The data in both datasets with EF < 40 and EF ≥ 40 were mixed and were difficult to segregate from each other.

The model achieved an AUC value of 0.95 (95% confidence interval [CI]: 0.947 to 0.953) on the dynamic EchoNet-Dynamic dataset with increasing training (Figure 1B). Moreover, the stable convergence proved that training was sufficient. An AUC value of 1 (95% CI: 1 to 1) was achieved on the local independent validation dataset (Figure 1C). These results suggest that the model had excellent classification ability on the dynamic ultrasound dataset. The complete model structure can be obtained from Figure S1.

#### Static recognition capability of the model

**Figure 2:**
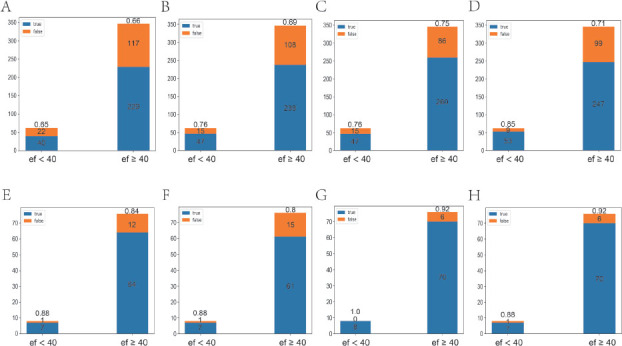
Classification effect of the model on the static dataset. A–D. r2plus1d-Pan1 classification effects when 1, 2, 4, and 8 static images from the same echocardiogram were randomly input, respectively. E–H. r2plus1d-Pan2 classification effects when 1, 2, 4, and 8 static images from the same echocardiogram were randomly input, respectively.

The classification accuracy (ACC) values of the r2plus1d-Pan1 model were 0.66, 0.70, 0.75, and 0.74 with 1, 2, 4, and 8 images, respectively. By contrast, the classification accuracy values of the r2plus1d-Pan2 model were 0.85, 0.81, 0.93, and 0.92 with 1, 2, 4, and 8 images, respectively. The classification accuracy of the r2plus1d-Pan2 model was significantly higher than that of the r2plus1d-Pan1 model (*P* = 0.003, Figure 2). This is because the r2plus1d-Pan2 model uses 80% of the total data from the CAMUS dataset for training and is more "adapted" to static ultrasound data than the r2plus1d-Pan1 model. This suggests that even if the compatibility of dynamic and static ultrasound data is achieved, further research is required to understand the practical applications of the r2plus1d-Pan2 model as it would require a large amount of co-learning of both types of data.

### Comparison with human experts

Because ultrasonography relies on the operator’s experience, the four most senior physicians were selected and their results were compared with those of AI. After four sequential classifications using 1, 2, 4, and 8 images, the optimal performance values of the human expert in each classification task were 0.82, 0.83, 0.85, and 0.88, respectively, which were not significantly different from the performance of AI (*P* = 0.34). Additionally, the median performance values of the expert group were 0.655, 0.680, 0.685, and 0.805, respectively, which were significantly lower than those of the AI group (*P* = 0.003, Table 1).

**Table 1:** Comparison of the classification accuracy between highly qualified experts and artificial intelligence based on static images.

	One image	Two images	Four images	Eight images
H3+_1	0.55	0.55	0.40	0.55
U3+_1	0.32	0.38	0.54	0.75
U3+_2	0.76	0.83	0.85	0.88
U3+_3	0.82	0.81	0.83	0.86
AI	0.85	0.81	0.93	0.92

H3+: specialists from the cardiology department with more than 3 years of experience (one expert meets the requirements); U3+: specialists from the ultrasound department with more than 3 years of experience (three experts meet the requirements).

**Figure 3:**
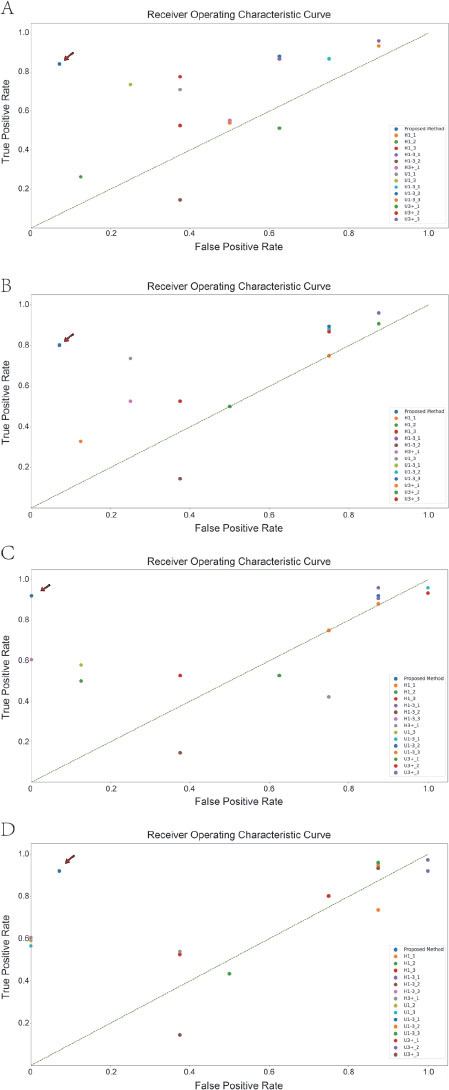
Comparison of the classification results between all human experts and artificial intelligence on static data. A-D. Classification results using 1, 2, 4, and 8 graphs at a time. Artificial intelligence models are indicated using arrows. H1: specialists who have been working in cardiology specialties for less than 1 year; H1-3: specialists who have been working in cardiology specialties for 1–3 years; H3+: specialists who have been working in cardiology for more than 3 years; U1: specialists who have worked in ultrasound for less than 1 year; U1-3: specialists who have worked in ultrasound for 1–3 years; U3+: specialists who have worked in the ultrasound specialty for more than 3 years.

The results of each classification by the experts and AI were visualized (Figure 3). The point where AI was located was closest to the coordinates (0,1) .

## Discussion

The objectification, standardization, and paper-based nature of cardiac ultrasonography are necessary for clinical work.^[14]^ For example, a patient with HF needs to travel from an area of scarce medical resources to a relatively abundant area. In this case, the patient could carry only a paper ultrasound report with some printed images. However, upon arrival at the destination, the patient often does not have immediate access to such services because the demand for cardiac ultrasound examinations at each medical unit is greater than the supply. As a result, physicians often have to rely on available examination reports for processing and wait for the results of their own unit’s ultrasound examinations. Because ultrasound is highly influenced by operator subjectivity, results given by different operators can vary, thereby resulting in inappropriate medical measures during the early stages of treatment.^[2,3]^

The information deficit caused by missing dimensions is a major obstacle in achieving compatibility between static and dynamic data. If dynamic video is considered as a series of consecutive frames, it contains information as both frames and sequences. A frame could contain spatial information at that moment, whereas a sequence would contain the temporal variation of this spatial information. However, static images lose temporal information. The necessity of temporal information for accurate action recognition and whether the static frames of a sequence contain this information is still debated.^[13,15]^

The degradation of classification caused by missing temporal information can be compensated for using model restructuring and training with multiple data types. The classification accuracy of r2plus1d-Pan2 is significantly higher than that of r2plus1d-Pan1 (*P* = 0.003), which suggests that the classification capacity of the model could be significantly improved by supplementing the relevant training data. Compared with AI models that are compatible with either dynamic or static ultrasound data, the models that are compatible with both static and dynamic data have several advantages. They could be used for a wider range of applications in paper-based examination reports (static) and electronic device (dynamic) settings. Better standardization of cardiac ultrasound examinations reduce the subjective influence of the operator and variations in examination results in different regions with different levels of healthcare resources. They compensate for the reduced classification effect due to the missing temporal information while making the conversion from dynamic to static data.

However, there are some limitations to the practical application of the model compatible with both static and dynamic data. Achieving compatibility with multimodal data would require a large amount of data to train and further improve the performance of the AI model and higher training costs. AI-assisted diagnosis and treatment models can only provide reference advice to clinicians but cannot replace human experts in diagnosis and treatment. The actual environment in which the model will be applied would be far more complex than the laboratory environment. This, in turn, requires further training to improve stability.

There are several highlights in this study. First, this is the first deep learning model compatible with dynamic and static ultrasound, and it can accurately perform HF diagnosis. The model outperformed most human experts from the National Cardiovascular Centers who have worked in ultrasound and cardiac specialties for more than 3 years. Second, the proposed model is the largest trained AI ultrasound diagnostic model. Its training and human-expert comparisons were performed using a multi-center study approach, thus confirming the stability of the results.

This study has some limitations. First, larger-scale multi-center data are needed, including more application populations, data types, and data collection devices. Second, the model needs further lightweighting because it is too large, with a file size of 57.2 GB.

In this study, a new deep spatio-temporal convolution model was constructed. The proposed model accurately identified patients with HF with reduced EF (< 40%) using dynamic or static cardiac ultrasound images. The model outperformed most senior specialists in diagnostic performance, and thus, it can improve overall HF diagnosis. Moreover, it allows patients to be referred and reexamined with a portable static ultrasound report, thereby reducing medical risk and saving healthcare resources.

## Supplementary Material

Supplementary Materi

## References

[1] Shen L, Jhund PS, Petrie MC, Claggett BL, Barlera S, Cleland JGF (2017). Declining risk of sudden death in heart failure. N Engl J Med.

[2] Steinmetz P, Oleskevich S, Lewis J (2016). Acquisition and long-term retention of bedside ultrasound skills in first-year medical students. J Ultrasound Med.

[3] Elison DM, McConnaughey S, Freeman RV, Sheehan FH (2020). Focused cardiac ultrasound training in medical students: Using an independent, simulator-based curriculum to objectively measure skill acquisition and learning curve. Echocardiography.

[4] Brooks PM, Lapsley HM, Butt DB (2003). Medical workforce issues in Australia: “tomorrow’s doctors--too few, too far”. Med J Aust.

[5] Gorman DF, Brooks PM (2009). On solutions to the shortage of doctors in Australia and New Zealand. Med J Aust.

[6] Zhang Y, Coyte PC (2020). Inequality of opportunity in healthcare expenditures: evidence from China. BMC Health Serv Res.

[7] Kim SJ, Peterson CE, Warnecke R, Barrett R, Glassgow AE (2020). the uneven distribution of medically underserved areas in Chicago. Health Equity.

[8] Ouyang D, He B, Ghorbani A, Yuan N, Ebinger J (2020). Video-based AI for beat-to-beat assessment of cardiac function. Nature.

[9] Leclerc S, Smistad E, Pedrosa J, Ostvik A, Cervenansky F, Espinosa F (2019). Deep Learning for segmentation using an open large-scale dataset in 2D echocardiography. IEEE Trans Med Imaging.

[10] Salem Omar AM,, Shameer K, Narula S, Abdel Rahman MA, Rifaie O, Narula J (2018). Artificial intelligence-based assessment of left ventricular filling pressures from 2-dimensional cardiac ultrasound images. JACC Cardiovasc Imaging.

[11] Voelker R (2020). Cardiac ultrasound uses artificial intelligence to produce images. JAMA.

[12] Van der Maaten L, Hinton G (2008). Visualizing data using t-SNE. Journal of Mach Learn Res.

[13] Tran D, Wang H, Torresani L, Ray J, LeCun Y, Paluri M (2018). A closer look at spatiotemporal convolutions for action recognition. Proceedings of the IEEE conference on Computer Vision and Pattern Recognition.

[14] Kossaify A (2021). Quality assurance and improvement project in echocardiography laboratory: the pivotal importance of organizational and managerial processes. Heart Views.

[15] Qiu Z, Yao T, Mei T (2017). Learning spatio-temporal representation with pseudo-3d residual networks. Proceedings of the IEEE International Conference on Computer Vision.

