# Supplementary figures and images for: A generalized deep learning model for heart failure diagnosis using dynamic and static ultrasound
Source: J Transl Int Med. 2023 Jul 5;11(2):138–44. doi: 10.2478/jtim-2023-0088 (PMC10680380; doi:10.2478/jtim-2023-0088)

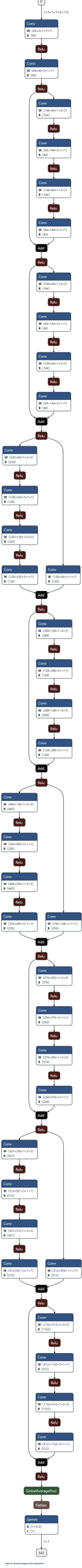

Figure S1. Structural diagram of the r2plus1d-Pan.

Supplement: Supplementary file 1 — Supplementary Materi [file jtim-2023-0088_SM.pdf]
